# “Appropriateness of colonoscopy according to EPAGE II in a low resource setting: a cross sectional study from Sri Lanka”

**DOI:** 10.1186/s12876-018-0798-7

**Published:** 2018-05-29

**Authors:** Yasara Samarakoon, Nalika Gunawardena, Aloka Pathirana, Sumudu Hewage

**Affiliations:** 1 National Cancer Control Programme, No.555, Public Health Building Complex, Elvitigala Mawatha, Colombo, 5 Sri Lanka; 2 World Health Organization Country Office for Sri Lanka, No. 5, Anderson road, Colombo, 5 Sri Lanka; 3 Department of surgery, Faculty of Medical Sciences, University of Sri Jayewardenepura, Sri Jayewardenepura, Sri Lanka

## Abstract

**Background:**

Due to finite resources, the clinical decision to subject a patient to colonoscopy needs to be based on the evidence, regardless of its availability, affordability and safety. This study assessed the appropriateness of colonoscopies conducted in selected study settings in Sri Lanka. In the absence of local guidelines, audit was based on European Panel on Appropriateness of Gastrointestinal Endoscopy II (EPAGE II) criteria.

**Methods:**

This cross-sectional study assessed consecutive patients who underwent colonoscopy between June to August 2015 at four main hospitals in Sri Lanka. Interviewer administered questionnaire and secondary data were collected by trained health staff. Indications were assessed according to EPAGE II criteria.

**Results:**

Out of 325 patients, male female proportions were 57.2 and 42.8%. Mean (SD) age was 54.9 (12.1) years. Colonoscopies were appropriate in 61.2% (95% CI 55.8–66.3), uncertain in 28.6% (95% CI 23.9–33.7) and inappropriate in 10.2% (95% CI 7.3–13.9). Colonoscopy to evaluate abdominal pain has highest percentage of inappropriateness of 10.0%. However, 9.5% of these colonoscopies revealed Colo-Rectal Cancer (CRC), reflecting differences in the profile of local CRC patients. Colonoscopies with appropriate or uncertain indications are three times more likely to have a relevant finding than inappropriate indications (42.5% vs. 18.2%; OR 3.32, 95% CI 1.33–8.3; *P* = 0.008).

**Conclusions:**

Majority of colonoscopies are appropriate. However, it cannot be neglected that every one in ten patients undergo inappropriate colonoscopy. Proportion of inappropriateness was highest for the indication of chronic abdominal pain, of which, 9.5% of patients were diagnosed with CRC. This may reflect the different profile of local CRC patients in terms of symptom manifestation and other characteristics. In conclusion, the authors recommend formulation of national guidelines for colonoscopy indications based on current best evidence and local patient profile. Use of such prepared local guidelines will improve the efficient use of finite resources.

## Background

Using finite resources to deliver infinite health needs of people is a problem faced by health care decision makers in both Low-Middle and High-Income countries. Clinicians can help to manage this situation better by adhering to evidence-based guidelines in investigating and managing patients. This will ensure the clinician’s decisions are effective and efficient. Availability, affordability and safety of an investigation are all important but secondary factors that needs to be considered in ordering an investigation.

The frequency of lower endoscopic procedures performed has gradually increased during the last decade [1]. A recent survey in the United States (US) found that the number of colonoscopies performed had risen three to four times between 1998 and 2004 [2]. The pattern in the Europe is found to be similar [3]. Improved efficacy of colonoscopy in colorectal cancer (CRC) screening and quality improvements in some aspects of the procedure like conscious sedation and safety of patients are some of these commonly mentioned reasons for the increase number of colonoscopies in the developed countries [1]. In addition, establishment of open-access endoscopy units in the US and Europe where any physician may request an endoscopic procedure is said to have led to an increase in inappropriate referrals and number of endoscopies performed in the western world [1, 4].

The European Panel Appropriateness Gastrointestinal Endoscopy (EPAGE-I) was founded in 1999 with the main aim of streamlining the Gastro-Intestinal endoscopies. EPAGE-I criterion contains 12 main indications for colonoscopy, with 309 different clinical scenarios. Each clinical situation is scored from 1 to 9. Scores reflect the appropriateness of the colonoscopy as appropriate 7–9; uncertain 4–6 and inappropriate 1–3 [5]. Updated version of the EPAGE-I criteria has been published recently as EPAGE-II [6]. A clinical validation study done on EPAGE II criteria concluded that significant lesions were more prevalent in appropriate colonoscopies than in those considered inappropriate (OR = 1.95, 95% CI: 1.2–3.1, *P* < 0.005) [7].  Application of such criteria reduces the rates of inappropriateness and more importantly, decrease the rate of missed significant lesions [8, 9]. However, it should be noted that these criteria are imperfect, and there is a chance of detecting a significant lesion in about 30% of inappropriate colonoscopies due to incidental finding of asymptomatic lesions [10].

In Sri Lanka, a national registry is not available for patients who undergo colonoscopies, but data from isolated institutions clearly show an increasing trend. Total number of colonoscopies from the Colombo South Teaching Hospital increased from 785 in 2015 to 947 in year 2016. Absence of studies on evaluation of the appropriateness of colonoscopy published to date in Sri Lanka has left a gap on essential knowledge in the reasons behind the upward trend of colonoscopies in Sri Lanka This gap needs to be fulfilled if we are to improve the cost-effectiveness of the procedure and quality of care, both of which will lead to improved universal health coverage in a country. With this background, we conducted this study to evaluate the appropriateness of colonoscopies performed in selected study settings according to EPAGE-II and to assess predictive factors including appropriateness of the colonoscopy for positive findings.

## Methods

### Study settings

This cross-sectional study was carried out in specialized gastro-intestinal (GI) colonoscopy units at the five main tertiary care hospitals in Sri Lanka. Each of these GI endoscopy unit is under the supervision of one or more consultant gastro-enterologist or general surgeon.

### Subjects

Every consecutive patient who was eligible and underwent colonoscopy during the time period of three months from June 2015 to August 2015 at these study settings was selected. Those who were undergoing regular follow-up colonoscopy, those with incomplete colonoscopies and those with inadequate bowel preparation were excluded. Informed written consent was obtained from all participants while the ethical approval for the study was obtained from the Ethical Review Committee of the University of Colombo under the protocol no EC-15-078.

### Data collection

Information regarding indication of the colonoscopy were obtained from the colonoscopy request forms for out-patients and from the Bed Head Tickets (BHT) for inward patients. In addition, patients were interviewed before the procedure to capture the history of the symptoms and signs and their medical records were used to extract information required for EPAGE criterion. All the information was collected by trained health professionals (medical officers and nurses) using a questionnaire prepared specifically for this study. Study participants were followed up to acquire colonoscopy findings and the histo-pathological diagnosis of the lesions when necessary. This collected information were then fed to evaluate the appropriateness of the clinical decision to subject the patient to colonoscopy according to EPAGE II criterion.

### Statistical review

The main outcome variable of appropriateness of colonoscopy was determined according to the EPAGE II criterion. This criterion classifies appropriateness into 3 categories based on the total marks: appropriate (≥7), uncertain (4–6) and inappropriate (> 3) [4]. These ‘appropriate’ and ‘uncertain’ indications were amalgamated together and were evaluated against ‘inappropriate’ indications in assessing the associated factors using the Chi squared test. A descriptive analysis was performed on the patients’ socio-demographic data, clinical indication of colonoscopy, results of the procedure and histological diagnosis where appropriate. Means and standard errors were used to describe continuous data, while rates and proportions were used for categorical data. Appropriateness level was determined to individual indication. A bivariate analysis was performed for the appropriateness of colonoscopy, dichotomized by the cut-off value of 7on the EPAGE-II scoring system, with patient factors and diagnosis relevance. Differences between groups were assessed using Chi squared test or Fisher’s exact test to compare categorical variables. Student’s t test or Mann-Whitney U test was used for continuous variables, depending on the normality of their distribution. The level of significance was considered at 5% in all contrasts. Subsequently, multivariate logistic regression was performed to assess variables associated with appropriateness, considering the variables which were identified to be significant at a *p* value ≤0.20 or clinically relevant variables according to literature as independent variables.

## Results

We could achieve a 100% response rate from patients from both state and private sector patients. A total of 325 colonoscopies were included over the study period out of which, 239 (73.5%) were from state hospitals and 86 (26.4%) were from the private hospital. The study group consisted of 186 (57.2%) males and 139 (42.8%) females. The mean (SD) age was 54.9 (12.1). Percentage who has never actively smoked was 70.8%, while 66.5% had never taken alcohol. None was follow-up colonoscopies for diagnosed CRC patients while 22 (6.8%) patients had a family history of CRC among the first degree relatives. Family history of Breast cancer, Ovarian cancer and Uterine cancer were reported in 14 (4.3%), 1 (0.3%) and 7 (2.2%) of patients. The descriptive data for the study population is shown in Table 1.

**Table 1:** Descriptive data for the study population (*N* = 325)

Variable	Number	Percentage (%)
Sex		
Male	186	57.2
Female	139	42.8
Age		
30–39 years	37	11.4
40–49 years	69	21.2
50–59 years	97	29.8
60–69 years	79	24.3
≥ 70 years	43	13.2
Race		
Sinhala	290	89.2
Tamil	17	5.2
Moor	16	4.9
Burger	2	0.6
Religion		
Buddhism	210	64.6
Hindu	12	3.7
Islam	16	4.9
Christian	87	26.8
Living area		
Urban	260	80.0
Rural	65	20.0
Educational status		
No education	3	0.9
Grade 1- Advanced Level	225	69.2
Advanced Level completed	69	21.2
Technical/Professional/Diploma	13	4.0
University education or above	15	4.6
Marital status		
Unmarried	27	8.3
Married/living together	262	80.6
Marries and living separately	5	1.5
Divorced or widowed	31	9.5
Family history of colorectal cancer among first degree relatives		
Yes	22	6.8
No	303	93.2
Family history of other cancers among first degree relatives		
Breast cancer	14	4.3
Ovarian cancer	1	0.3
Uterine cancer	7	2.2
Prostate cancer	2	0.6
Other types of cancers	9	2.8
Smoking status		
Never actively smoked	230	70.8
Active smoking in the past	50	15.4
Active smoking past and present	35	10.8
Passive smoking only	10	3.1

Among the 325 patients who underwent colonoscopy, three commonest indications were rectal bleeding (*n* = 110, 33.8%), change in bowel habits (*n* = 79, 24.3%) and unexplained chronic abdominal pain (*n* = 61, 18.8%) sequentially. Half of colonoscopies (*n* = 168, 51.7%) ended up as normal studies. Polyps were detected and sampled in 98 (30.1%) patients, but none of them were diagnosed as adenomatous polyps histologically. Therefore, all polyps were categorized under normal study. Table 2 displays colonoscopy findings against the indication.

**Table 2:** Colonoscopy findings against the indication (*N* = 325)

Colonoscopy finding						
	Normal study *n* (%)	Colo-rectal Carcinoma *n* (%)	Haemorrhoides *n* (%)	Anal fissure *n* (%)	Diverticular disease *n* (%)	Total *n* (%)
Indication for colonoscopy						
Rectal bleeding	52 (16.0)	58 (17.8)	19 (5.8)	0 (0.0)	1 (0.3)	130 (40.0)
Altered bowel habits	56 (17.2)	34 (10.5)	2 (0.6)	0 (0.0)	0 (0.0)	92 (28.3)
Abdominal pain	39 (12.0)	31 (9.5)	4 (1.2)	0 (0.0)	0 (0.0)	74 (22.8)
Screening	19 (5.8)	5 (1.5)	0 (0.0)	0 (0.0)	0 (0.0)	24 (7.4)
Lump at anus	1 (0.3)	0 (0.0)	2 (0.6)	1 (0.3)	0 (0.0)	4 (1.2)
Loss of weight	1 (0.3)	0 (0.0)	0 (0.0)	0 (0.0)	0 (0.0)	1 (0.3)
Total *n* (%)	168 (51.7)	128 (39.4)	27 (8.3)	1 (0.3)	1 (0.3)	325 (100.0)

As detailed in Table 3, colonoscopies were evaluated as appropriate in 61.2% of the patients (95% CI 55.8–66.3), uncertain in 28.6% (95% CI 23.9–33.7) and inappropriate in 10.2% (95% CI 7.3–13.9) according to EPAGE II criteria. Rectal bleeding and changes in bowel habits were the symptoms which presented the highest percentage of appropriateness, 65.0 and 30.0%respectively, while lump at anus presented the lowest appropriateness of 0.0%. Abdominal pain rescored the highest percentage of inappropriateness of 9.5%, as a symptom for colonoscopy. 89.7% of opportunistic screening was appropriate according to EPAGE criteria, while 6.5% were uncertain and 3.7% were inappropriate. Appropriateness of colonoscopy significantly increased in relation to age from 52.6% in patients more than 50 years of age to 8.6% in patients less or equal to 50 years of age (chi square 89.5; *p* < 0.001). Appropriateness was also evaluated for symptomatic against screening colonoscopies. The clinical decision to subject some symptomatic patients to colonoscopy was the clinician’s opinion of the risk of the patient for CRC being high rather than the symptom. Such patients identified through their medical records and were categorized under the ‘opportunistic screening’ category rather than the ‘symptomatic category’. This resulted in total of opportunistic screening colonoscopies to be 107 (32.9%) in the Table 4, as opposed to 24 (7.4%) in the Table 3. Indication being symptomatic was significantly associated with appropriate colonoscopy rather than opportunistic screening (*p* < 0.001). Classification of colonoscopy appropriateness according to EPAGE II criteria against selected characteristics is shown in Table 3, while the factors associated with appropriate colonoscopy according to EPAGE II criteria is shown in Table 4.

**Table 3:** Classification of colonoscopy appropriateness according to the EPAGE II criteria against selected characteristics of the patient and indication. EPAGE II criteria percentages for each factor are shown per row

		Classification according to EPAGE criteria			
	Total *N* = 325 (%)	Inappropriate ≤3 *n* = 33 (10.2%)	Uncertain 4–6 *n* = 93 (28.6%)	Appropriate ≥7 *n* = 199 (61.2%)	*p* value
Age					< 0.001
≤ 50 years	102 (31.4)	29 (28.4)	45 (44.1)	28 (27.4)	
> 50 years	223 (68.6)	4 (1.8)	48 (21.5)	171 (76.6)	
Sex					0.41
Women	163 (50.1)	19 (11.6)	42 (25.7)	102 (62.6)	
Men	162 (49.9)	14 (8.6)	51 (31.5)	97 (59.9)	
Type of colonoscopy					< 0.001
Symptomatic	218 (67.0)	27 (12.4)	86 (39.4)	103 (47.2)	
Screening	107 (32.9)	4 (3.7)	7 (6.5)	96 (89.7)	
Indication for symptomatic colonoscopies (*n* = 218)					< 0.001
Rectal bleeding	96 (44.0)	4 (4.1)	25 (26.0)	67 (69.8)	
Altered bowel habits	44 (20.2)	4 (9.0)	9 (20.4)	31 (70.4)	
Abdominal pain	31 (14.2)	10 (32.2)	21 (65.6)	0 (0.0)	
Other	47 (21.5)	11 (23.4)	31 (65.9)	5 (10.6)

**Table 4:** Factors associated with appropriate colonoscopy according to EPAGE II criteria

Factor	OR	95% Confidence interval for OR	*P* value
Age			
≥ 50 years	8.52	**4.90–14.82**	< 0.001
< 50 years			
Sex			
Male	0.95	0.56–1.58	0.835
Female			
Institution			
NCIM	.951	0.49–1.83	.881
NHSL	2.727	**1.19–6.24**	.018
CSTH	.653	0.13–3.14	.595
CNTH	1.076	0.47–2.46	.862
Private Hospital			

NCIM – National Cancer Institute – Maharagama

NHSL – National Hospital of Sri Lanka

CSTH – Colombo South Teaching Hospital

CNTH – Colombo North Teaching Hospital

95% Confidence Interval are in bold

Multivariate logistic regression revealed that appropriateness of colonoscopy was significantly associated with the age being more than 50 years (OR = 8.5; *p* < 0.001; 95% CI 4.9–14.8). One out of the five institutions, the National Hospital of Sri Lanka, was also found to be significantly associated with the appropriateness of colonoscopy (22OR = 2.7; *p* = 0.18, 95% CI 1.2–6.2). Sex of the patient or the indication of colonoscopy being screening or symptomatic was not significantly associated with the appropriateness according to EPAGE II criteria.

## Discussion

Every single unnecessary investigation or procedure is afforded at the cost of another necessary health service or an item. Limiting investigations to those who require it also helps to reduce the work load of the health care team, which will in return lead to improvement in the quality of care and low cost. EPAGE II is a valid set of criteria which has been shown to be valid to identify appropriate gastrointestinal endoscopy [6, 11, 12].

Rectal bleeding ranked number one as the indication for colonoscopy (*n* = 130; 40%), followed by altered bowel habits (*n* = 92; 28.3%) and abdominal pain (*n* = 74; 22.8%). These indications were similar to common indications found in other European studies as well [12–14]. However, opportunistic screening for CRC was done only in 24 (7.4%) out of the 325 studied. Half of colonoscopies (51.7%) were normal studies, while colorectal carcinoma was found in 39.4% and haemorrhoides in 8.3% of study participants. Out of 128 patients who were diagnosed to have CRC, 58 (17.8%) had presented with rectal bleeding, 34 (10.5%) with altered bowel habits and 31 (9.5%) with abdominal pain. Only 5 (1.5%) of CRC patients were detected through opportunistic screening.

The majority of the indications for colonoscopy are appropriate according to the EPAGE II criteria (61.2, 95% CI 55.8–66.3%). However, it should be noted that 28.6% (95% CI 23.9–33.7%) of uncertain and 10.2% (95% CI 7.3–13.9%) of inappropriate criteria cannot be neglected. Studies from all over the world have reported percentages within the range of for appropriateness of colonoscopy. Highest reported thus far according to EPAGE II evaluation is 80.4% [14], while the lowest (26%) is recorded from a multi-country study which involved 21 endoscopy centers from 11 countries [15]. One study from Ivory Coast, Africa found out an appropriateness percentage to be 40% [16], which is below our finding. Scarcity of regional and local literature seriously hinders evaluating the position of Sri Lanka relative to other South Asian countries which are socio-economic and health system wise similar. However, our study finding of appropriateness of colonoscopy according to EPAGE II criteria of 61.2% is in par with the findings from most European studies [16–18]. But when the proportions of inappropriateness are considered, values as high as 27% have been reported in a multicentric study in 11 European countries [16]. European countries which show higher percentages of inappropriate indications than that from our study include Switzerland at 13–27% [19, 20–22] and Spain at 23–31% [22]. However, this proportions have improved after 2009, when the evaluation is done using EPAGE II criteria. Spanish studies conducted after 2009 records a percentage of 10.5–17.5% inappropriate colonoscopy [13, 23], which is similar to this study. Even though the percentage estimated by this study is low, the number cannot be neglected when the cost borne by the health system for a colonoscopy is considered. Based on this evidence, the clinicians need to consider the indication for colonoscopy more carefully.

Likewise, symptomatic colonoscopy was found to be more appropriate than opportunistic screening colonoscopies (*p* = < 0.001). As described previously, opportunistic screening group consisted of patients who were subjected to colonoscopies purely for screening purposes without any symptom and also symptomatic patients. The clinical decision to subject this proportion of symptomatic patients to colonoscopy not being their symptom per se, but also the clinician’s opinion that the patient was at high risk of developing CRC was the rationale to include them in the opportunistic screening category even though they presented with symptoms. This significant association strengthen the evidence of subjecting a patient to colonoscopy based on guidelines.

Out of the 218 symptomatic colonoscopies, more than half had been performed to evaluate rectal bleeding (40.4%) and altered bowel habits (20.2%), with least percentages of inappropriateness of 4.1 and 9.0% respectively. In contrast, abdominal pain assessment through colonoscopy does not seem to be very useful according to EPAGE II. Total of 31 colonoscopies done to assess chronic abdominal pain is categorized as inappropriate in 32.2% (*n* = 10) and the rest 21 (65.5%) fell into uncertain category. None of the colonoscopy was graded as appropriate (Table 3). However, results of 9.2% of colonoscopies performed to evaluate chronic abdominal pain have turned out to be CRC, which is a higher proportion compared to other studies (7,8). This might indicate that some presenting symptoms of CRC patients in Sri Lanka can be different to well recognized symptoms. Therefore, it is important that characteristics of local patient population is systematically studied, especially with regard to their symptom profile, and local indications for colonoscopy are adjusted to suit the local epidemiological picture. Future research should explore more on this avenue and the findings should be used in making local guidelines for colonoscopy indications. Use of such local guidelines which are formulated based on local epidemiological and symptom profile would be more suitable in auditing colonoscopy appropriateness in Sri Lanka rather than using international guidelines. However, limitations inherited to cross-sectional studies in an out-patient clinic set-up like inability to randomly assign patients, which could introduce a selection bias, should be borne in mind in planning future studies.

Patients with appropriate or uncertain indications based on EPAGE II criteria are found to be three times more likely of having a relevant colonoscopy finding than those with inappropriate indications (42.5% vs. 18.2%; OR 3.32, 95% CI 1.33–8.3; *P* = 0.008). This evidence strongly suggests that adherence to guidelines in performing colonoscopies is more likely to improve efficient use of limited resources, while improving patient safety and the quality of the health services delivered to the patients.

## Abbreviations

BHT: Bed Head Ticket
CRC: Colo-Rectal Cancer
EPAGE: European Panel on Appropriateness of Gastrointestinal Endoscopy
GI: Gastro-intestinal
